# Bi-Epitope SPR Surfaces: A Solution to Develop Robust Immunoassays

**DOI:** 10.1371/journal.pone.0112070

**Published:** 2014-11-05

**Authors:** Li Peng, Melissa M. Damschroder, Herren Wu, William F. Dall’Acqua

**Affiliations:** Department of Antibody Discovery and Protein Engineering, MedImmune, One MedImmune Way, Gaithersburg, MD, 20878, United States of America; National Central University, Taiwan

## Abstract

Surface plasmon resonance (SPR)-based immunoassays have numerous applications and require high affinity reagents for sensitive and reliable measurements. We describe a quick approach to turn low affinity antibodies into appropriate capture reagents. We used antibodies recognizing human ephrin type A receptor 2 (EphA2) and a ProteOn XPR36 as a model system. We generated so-called ‘bi-epitope’ sensor surfaces by immobilizing various pairs of anti-EphA2 antibodies using standard amine coupling. The apparent binding affinities to EphA2 and EphA2 detection sensitivities of the bi-epitope and ‘single-epitope’ surfaces were then compared. For all antibody pairs tested, bi-epitope surfaces exhibited an ∼10–100-fold improvement in apparent binding affinities when compared with single-epitope ones. When pairing 2 antibodies of low intrinsic binding affinities (∼10^−8^ M) and fast dissociation rates (∼10^−2^ s^−1^), the apparent binding affinity and dissociation rate of the bi-epitope surface was improved up to ∼10^–10^ M and 10^−4^ s^−1^, respectively. This led to an ∼100–200-fold enhancement in EphA2 limit of detection in crude cell supernatants. Our results show that the use of antibody mixtures in SPR applications constitutes a powerful approach to develop sensitive immunoassays, as previously shown for non-SPR formats. As SPR-based assays have significantly expanded their reach in the last decade, such an approach promises to further accelerate their development.

## Introduction

Surface plasmon resonance (SPR) is an optical technique used for characterizing molecular interactions. It offers real-time and label-free detection and quantitation of complex formation and dissociation over time, a key advantage over traditional methods such as fluorescent or radiolabeled binding assays. Since Liedberg *et al*. first immobilized an antibody on a sensor surface [1], [2], a variety of SPR-based immunoassays have been developed for detecting biomarkers or characterizing molecular interactions in medical diagnostics, drug discovery, food safety, and environmental monitoring [3]–[10].

Being the recognition component of many SPR immunoassays, antibodies play a key role in assay sensitivity and performance. Various antibody immobilization strategies have been developed and their impacts on performance compared [11]–[18], including (i) simple adsorption, (ii) covalent attachment using heterobifunctional cross-linkers, (iii) non-covalent coating using streptavidin/biotin, and (iv) oriented capture using Fc region-binding proteins (e.g. protein A or G) or affinity tags (e.g. polyhistidine-tag). Amongst these, non-covalent oriented capture methods usually result in the most functional surfaces. However, these are not desired in many applications, due to lesser surface stability and additional capturing steps needed after every regeneration cycle. Covalent immobilization approaches, such as amine coupling, yield the most stable surfaces. In particular, amine coupling usually entails protein immobilization *via* their amine groups to the 1-ethyl-3-[3-dimethylaminopropyl] carbodiimide (EDC) and N-hydroxysuccinimide (NHS)-activated carboxyl groups of sensor surfaces.

Antibody affinity ultimately dictates immunoassay sensitivities [19]–[21]. High affinity antibodies are preferred as they can rapidly produce the greatest number of stable immune complexes, therefore allowing for sensitive detection. Reliable immunoassays usually require affinity constants in the ∼10–^10^ M range [22]. When using a sandwich format, dissociation rates for the capturing antibodies typically need to be as slow as ∼10^−4^ s^−1^, thus allowing captured antigens from crude samples to remain bound for detection using a secondary antibody. However, antibodies rarely possess such high affinity or slow dissociation rates when directly derived from standard selection methods (e.g. phage or yeast libraries) or purchased as commercial reagents. Thus, new identification and/or affinity maturation campaigns are often needed [23]–[26]. Considering the time and effort required for such an endeavor, we sought a quick alternative approach to turn inferior antibodies with intrinsically low affinities and fast dissociation rates into robust capture reagents for immuno-SPR applications.

Mixing antibodies binding to different epitopes results in higher apparent binding affinities and assay sensitivities when compared with individual antibodies in solid-phase radioimmunoassays and enzyme-linked immunosorbent assays [27]–[30]. However, such an approach is still under-appreciated for SPR applications. Notably, it was reported that epitope synergy did not exist when antibodies were directly immobilized using amine coupling, and only occurred when captured through their Fc region (e.g. with protein G or anti-Fc antibodies) [28]. Such observations have limited the usage of so-called ‘bi-epitope’ sensors in SPR immunoassays. To explore this further, we have generated various bi-epitope sensor surfaces using standard amine coupling, and compared the corresponding apparent binding affinities and assay sensitivities with those measured using single-epitope surfaces. We used the multiplexed SPR instrument ProteOn XPR36 platform [31] and soluble human ephrin type A receptor 2 (EphA2) as a model system. EphA2 plays a key role in the formation and progression of various cancers, and its overexpression predicts poor prognosis in ovarian and esophageal carcinoma [32]–[34]. Furthermore, it was suggested that measuring soluble circulatory EphA2 levels could have utility in patients who may benefit from EphA2-based therapies [35].

## Materials and Methods

### Kinetics and affinity measurements on low density single-epitope surfaces

A ProteOn XPR36 instrument (Bio-Rad, Hercules, CA) was used to determine the kinetics of anti-EphA2 monoclonal antibodies (mAb) 3B10, 3F2, 3B2 and 1C1 (MedImmune) to human EphA2 (MedImmune). Standard amine coupling was used to immobilize each antibody (20 nM in 10 mM sodium acetate buffer, pH 5.0) to the EDAC/Sulfo-NHS activated surface of a GLC biosensor chip (Bio-Rad) at a density of ∼200–600 resonance units (RU) according to the manufacturer’s instructions. This corresponds to a density of ∼20–60 ng/cm^2^. EphA2 was prepared in phosphate buffered saline (PBS), pH 7.4, containing 0.005% Tween-20 (PBS-T) and injected at 100 µl/min for 200 s at concentrations of 100–6.25 nM and 20–1.25 nM (1∶2 dilutions) for antibodies 3B10/1C1 and 3F2/3B2, respectively. The dissociation phase was followed for 600 s. Surfaces were regenerated by injecting 10 mM glycine HCl, pH1.5, for 30 s. All sensorgram data were processed using ProteOn Manager 3.1 software (Bio-Rad) and fitted to a 1∶1 interaction model.

### Epitope binning

Epitope binning for mAbs 1C1, 3F2, 3B10 and 3B2 was performed using competition binding using a ProteOn XPR36 instrument. The ability of mAbs 1C1, 3F2, 3B10 and 3B2 to bind to immobilized human EphA2 in the presence of another antibody was assessed as follows: EphA2 was immobilized onto a GLC sensor chip at density level of ∼800 RU (∼80 ng/cm^2^) using standard amino coupling chemistry (see above). For a given antibody pair, the first antibody at a concentration of 1 µM in PBS-T buffer was injected at 30 µl/min for 150 s to the EphA2-immobilized surface. A mixture of this same antibody with the second antibody (1 µM each in PBS-T buffer) was then passed over the same surface. The extent of competition was derived from the additional binding detected. This process was repeated for all 6 antibody pairs (namely 3B10 *vs.* 3F2, 3B10 *vs.* 1C1, 3B2 *vs.* 3F2, 3B10 *vs.* 3B2, 1C1 *vs.* 3F2 and 1C1 *vs.* 3B2).

### Generation of high density single- and bi-epitope surfaces

In order to identify optimal conditions for immobilization, various parameters were tested, including pH (4.0–5.5) and antibody concentrations (50–150 nM). The most favorable condition was then identified. In summary, all immobilizations were performed at high density (>5,000 RU, or >500 ng/cm^2^), using an injection rate of 30 µl/min for 300 s, and 100 nM individual mAbs or mAb mixtures in 10 mM sodium acetate buffer, pH 5.0.

### Kinetics and apparent affinity measurements on high density single- and bi-epitope surfaces

EphA2 was prepared in PBS-T and injected at 100 µl/min for 150 s at concentrations of 10–0.625 nM (1∶2 dilutions) over high density bi- or single-epitope surfaces. The dissociation phase was followed for 600 s. Surfaces were regenerated by injecting 10 mM glycine HCl, pH 1.5, for 30 s. All sensorgram data were processed using ProteOn Manager 3.1 software and fitted to a 1∶1 interaction model.

### EphA2 detection using a sandwich SPR assay

A sandwich SPR assay was used to detect and quantify EphA2 in crude cell supernatants. EphA2 dilutions series (50 nM-2 pM) spiked in conditioned mammalian cell culture medium were injected at 30 µl/min for 400 s over the 3B10-1C1 bi-epitope or its corresponding 3B10 and 1C1 single-epitope surfaces. Captured EphA2 was then detected by injecting 100 nM of mAb 3B2 that recognizes a distinct EphA2 epitope at 100 µl/min for 150 s. Binding response was plotted against EphA2 concentrations.

## Results and Discussion

### Kinetics, affinity and epitope characterization of anti-EphA2 mAbs

**Figure 1:**
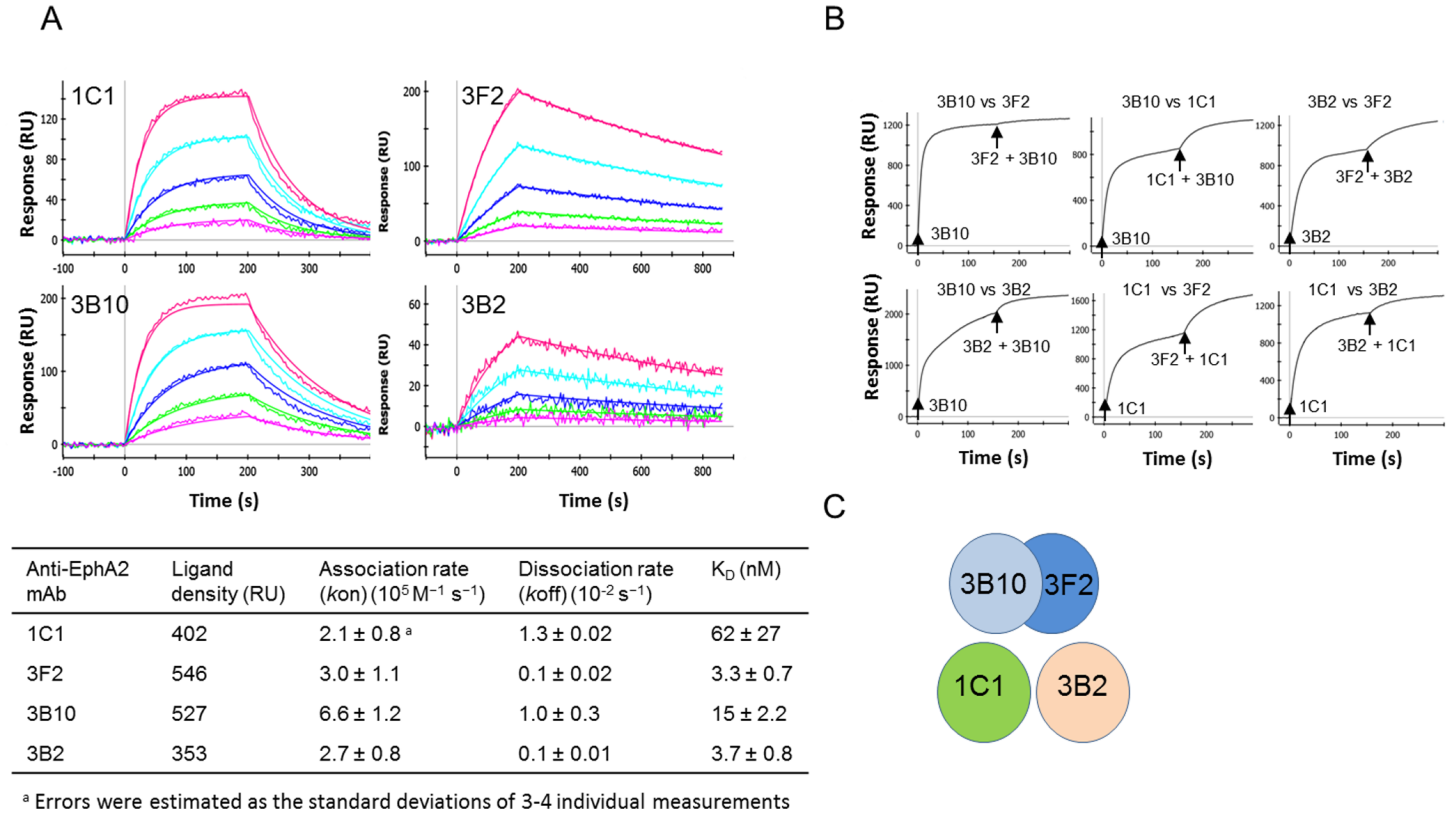
Binding and epitope characterization of various anti-EphA2 mAbs. (A) Binding kinetics of mAbs 1C1, 3F2, 3B10 and 3B2. Measurements were conducted using a ProteOn XPR36. Each antibody was immobilized at low density (∼200–600 RU or ∼20–60 ng/cm^2^) using amine coupling and EphA2 injected over the resulting surfaces. All 4 antibodies exhibit fast dissociation rates in the 10^−2^−10^−3^ s^−1^ range. (B) Epitope binning. Cross-competition binding studies between any pair of mAbs 1C1, 3F2, 3B10 and 3B2 was performed using a ProteOn XPR36 instrument. Injections are indicated by arrows. A response from the second injection indicated that each mAb in a given pair binds to a different epitope. (C) 3 distinct epitopes were identified, including 1 shared between mAbs 3B10 and 3F2.

Kinetics and affinity measurements, as well as epitope binning were performed on the 4 anti-EphA2 mAbs 1C1, 3F2, 3B10 and 3B2. All mAbs exhibited fast dissociation rates ranging from 1.3×10^−2^ to 1.0×10^−3^ s^−1^ (Figure 1A). These fast dissociation rates would prevent their usage as capture reagents in sensitive immunoassays. Additionally, mAbs 3B10 and 3F2 were found to recognize the same or largely overlapping epitope(s) (Figure 1B) and as such were not paired to generate a bi-epitope surface. mAbs 1C1 and 3B2 each recognized a distinct epitope from 3B10 and 3F2, as shown in Figure 1B. In summary, 3 distinct epitopes were identified (Figure 1C).

### Generation of high density single- and bi-epitope surfaces

**Figure 2:**
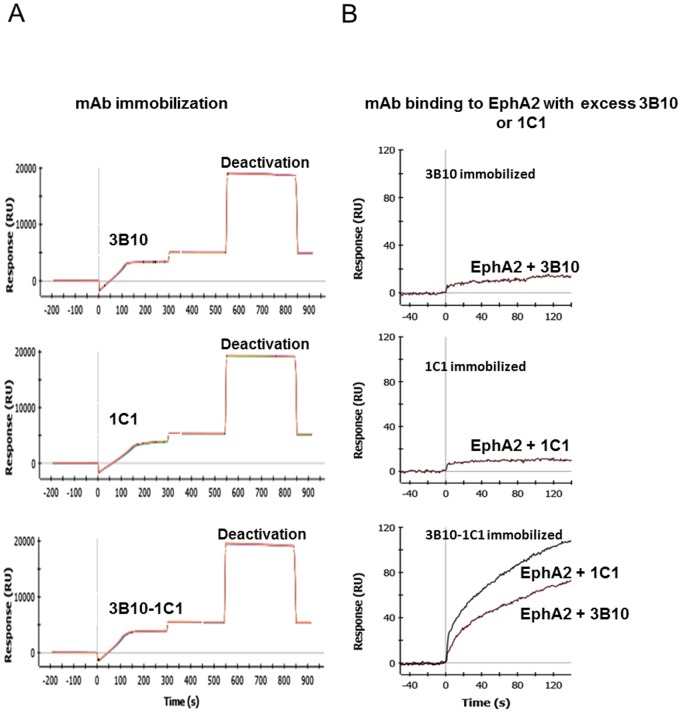
Generation and characterization of high density bi-epitope SPR sensor surfaces. (A) Immobilization sensorgrams of mAbs 3B10, 1C1 and 3B10-1C1 mixture. The immobilization profiles are comparable and yielded a high density surface (∼5,000–5,500 RU or ∼500–550 ng/cm^2^). (B) Confirmation of the co-existence of functional antibodies on the bi-epitope surfaces. Excess of mAbs 3B10 or 1C1 (1 µM) inhibited EphA2 binding to the single-epitope 3B10 or 1C1 surfaces, respectively, but not to the bi-epitope 3B10-1C1 surface.

To identify an optimal amine coupling condition, we tested various pH and mAb concentrations. We found that 100 nM IgGs in 10 mM sodium acetate buffer, pH 5.0, yielded the highest density levels (>5,000 RU or >500 ng/cm^2^) for all individual IgGs and their respective pairs (see Figure 2A for mAbs 3B10 and 1C1). The functionality of each antibody when immobilized together was assessed by injecting EphA2 (5 nM) in the presence of an excess of each individual mAb (1 µM). As shown in Figure 2B, the presence of excess mAb 3B10 or 1C1 inhibited the binding of EphA2 to the corresponding single-epitope surface, but not to the bi-epitope surface. This indicated that both mAb 3B10 and 1C1 were functional on the bi-epitope surface. The same observation was made for all mAb pairs (data not shown).

### Bi-epitope surfaces lead to substantial improvement in apparent dissociation rate and detection sensitivity

**Figure 3:**
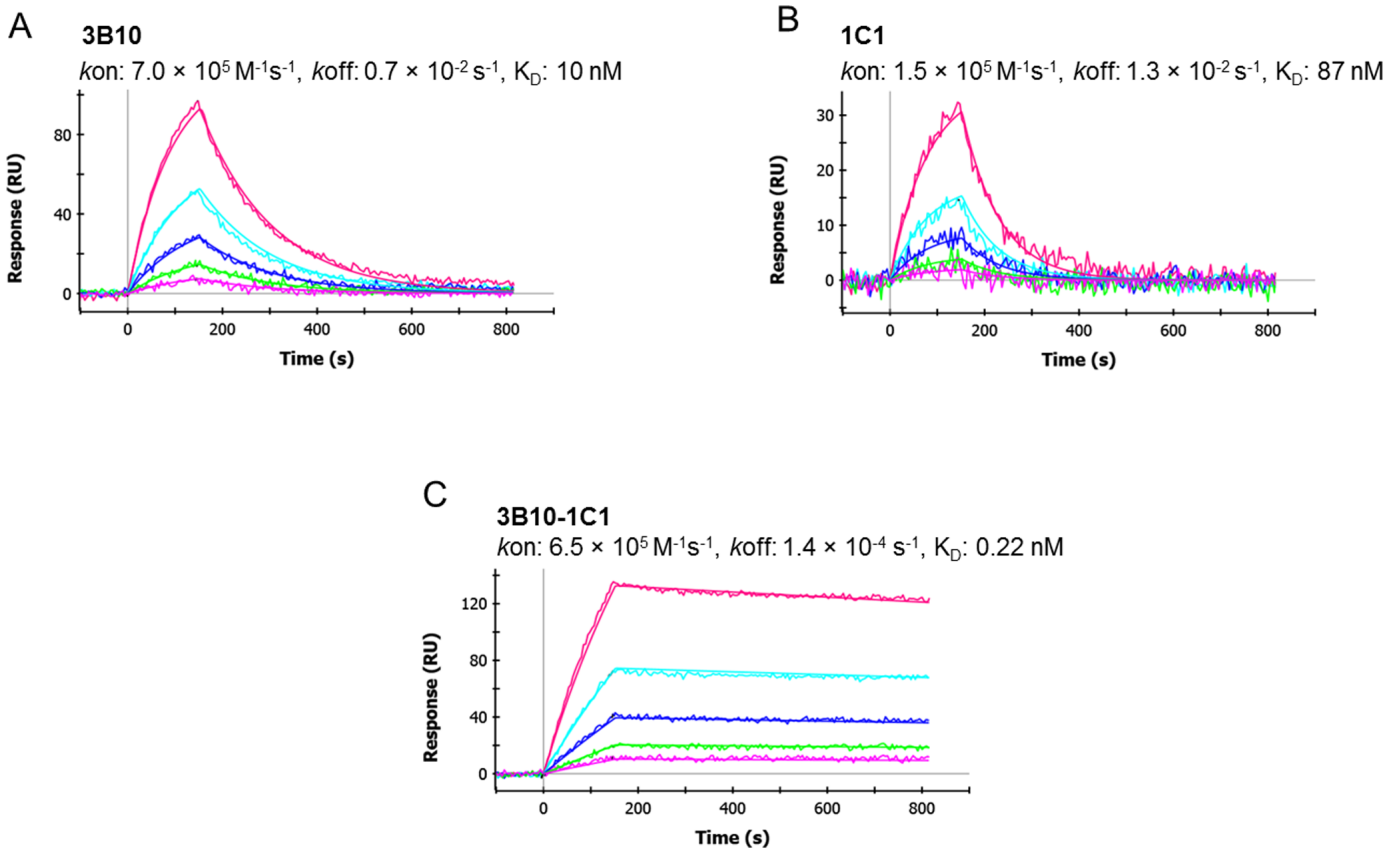
EphA2 binding to individual mAbs 3B10 (A), 1C1 (B) and corresponding mixture (C) immobilized at high density levels. When using the single-epitope high density surfaces, dissociation rates were fast and similar to that of the corresponding low density surfaces. Surfaces immobilized with the antibody pair allowed for an ∼100-fold increase in the apparent dissociation rate (∼10^−4^ s^−1^).

For any of the single-epitope high density surface, captured EphA2 quickly decayed with a similar dissociation rate to that of the corresponding low density surface (see Figure 3A–B with mAbs 3B10 and 1C1 as an example). In contrast, bi-epitope surfaces showed an ∼10–100-fold enhancement in their apparent dissociation rates (∼10^−4^–10^−5^ s^−1^, Table 1) when compared with that of the corresponding high density single-epitope surfaces. In particular, mixing mAbs 3B10 and 1C1, each possessing a very fast dissociation rate of ∼10^−2^ s^−1^, yielded a biosensor surface with an apparent dissociation rate of 1.4×10^−4 ^s^−1^ (Figure 3C and Table 1), an ∼100-fold improvement.

**Table 1:** Binding kinetics and affinities measured on high density bi-epitope sensors.

Anti-EphA2 mAbs	Ligand density (RU)^a^	Association rate (*k*on) (10^5 ^M^−1^ s^−1^)	Dissociation rate (*k*off) (10^−4 ^s^−1^)	K_D_ (nM)
3B10-1C1	5,502	6.5±1.2^b^	1.4±0.2	0.22±0.04
3B10-3B2	5,402	6.9±1.3	1.1±0.1	0.16±0.04
3F2-3B2	5,291	5.9±1.6	0.6±0.2	0.10±0.06
3F2-1C1	5,256	4.0±0.8	1.2±0.5	0.30±0.05
1C1-3B2	5,186	6.6±0.7	1.6±0.5	0.24±0.05

a Ligand density can also be expressed in ng/cm^2^, with 1 RU corresponding to 0.10 ng/cm^2^ [38], [39].

b Errors were estimated as the standard deviations of 3–4 individual measurements.

**Figure 4:**
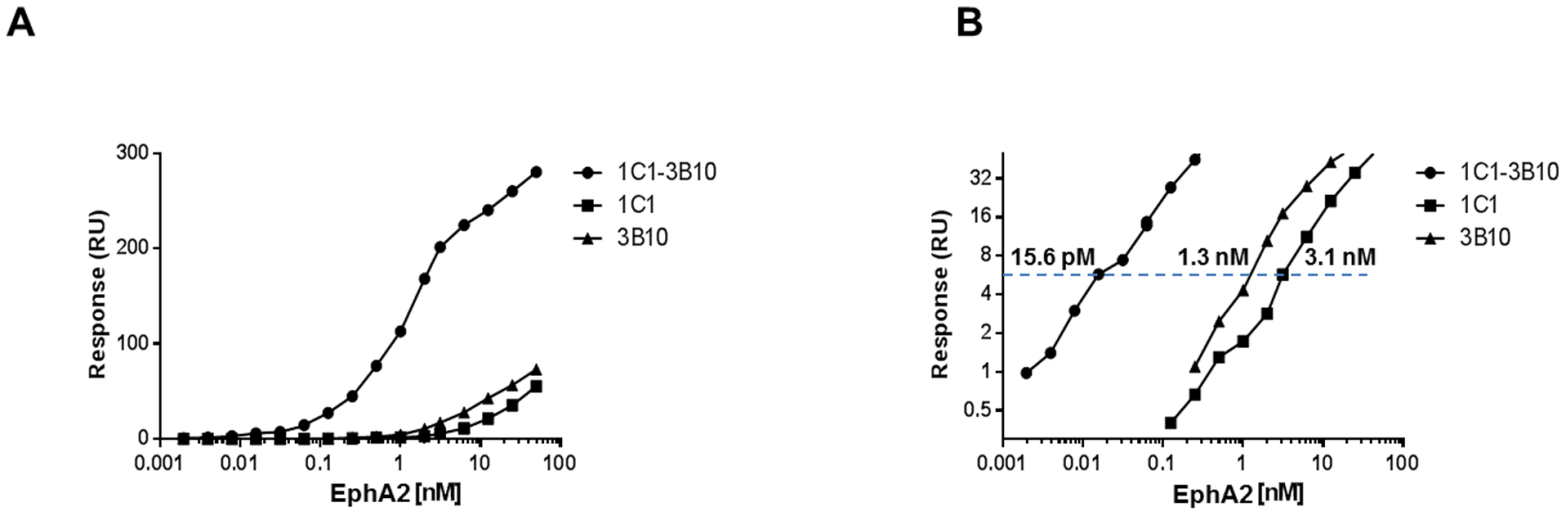
Pairing mAbs 3B10 and 1C1 results in enhanced EphA2 detection sensitivity in conditioned media. (A) Binding of detection antibody mAb 3B2 plotted against EphA2 concentrations. (B) Logarithmic scale display with binding signals in ∼0.3–30 RU (or ∼0.03–3 ng/cm^2^) range. The bi-epitope 3B10-1C1 surface detected the lowest EphA2 concentration (15.6 pM at a binding signal of 6 RU or 0.6 ng/cm^2^), an ∼100- and 200-fold improvement in detection limits when compared with the corresponding 3B10 (1.3 nM) and 1C1 (3.1 nM), respectively, single-epitope surfaces.

The tighter binding of bi-epitope surfaces led to a significant improvement in EphA2 detection sensitivity. We compared the respective ability of the bi-epitope 3B10-1C1 and single-epitope 3B10 and 1C1 surfaces to detect EphA2 in crude supernatant using a sandwich format. Under the same conditions, EphA2 spiked in conditioned mammalian cell culture medium was injected over the bi- or single-epitope surfaces, followed by the injection of a secondary antibody recognizing a different epitope on EphA2 (mAb 3B2; Figure 1C). Binding responses using the bi-epitope surface were much higher than that of the corresponding single-epitope surfaces (Figure 4A). A concentration as low as 15.6 pM EphA2 in crude supernatant could be detected with a binding signal of 6 RU (or ∼0.6 ng/cm^2^), an ∼100- and 200-fold improvement in detection limits when compared with the 3B10 (1.3 nM) and 1C1 (3.1 nM), respectively, single-epitope surfaces (Figure 4B). Thus, low affinities antibodies exhibiting fast dissociation rates can be turned into robust capture reagents to develop sensitive SPR immunoassays.

Although the amine coupling method is expected to result in the random orientation of antibodies on the sensor surfaces, we showed here that it can be quickly optimized to generate functional high density bi-epitope surfaces. Because of their large size (∼150 kDa) and flexibility, IgGs retain satisfactory ligand functionality regardless of random orientation upon immobilization. Indeed, their Fv domain (smallest antigen binding portion) is only ∼25 kDa, a small portion of the large ∼150 kDa IgG. Thus, a good proportion of Fvs will be spared from amine coupling modifications and thus remain functional. Additionally, IgGs are highly flexible molecules; their Fab arms can rotate by as much as 158° and the angles between Fab/Fc and Fab/Fab can range from 66–123° and 115–172°, respectively [36], [37]. Such flexibility likely also contributes to retain functionality in non-oriented coupling methods.

## Conclusion

This study introduces a quick method to turn low affinity antibodies into appropriate capture reagents for SPR-based immunoassays. Creating bi-epitope sensor surfaces using standard amine coupling leads to an ∼10–100-fold improvement in apparent binding affinities when compared with the individual antibody-coated surfaces. Antibodies exhibiting fast dissociation rates could be used to generate robust sensor surfaces. In the one example shown here, this led to up to an ∼100–200-fold improvement in antigen detection limits when compared with the corresponding single-epitope surface. Our approach extends to SPR applications the use of antibody mixtures in an effort to develop robust immunoassays.

## References

[1] Liedberg B, Nylander C, Lundstrom I (1983) Surface-Plasmon Resonance for Gas-Detection and Biosensing. Sensors and Actuators 4(2): 299–304.

[2] Liedberg B, Nylander C, Lundstrom I (1995) Biosensing with surface plasmon resonance–how it all started. Biosens Bioelectron 10(8): i–ix. 10.1016/0956-5663(95)96965-2 7576432

[3] Guidi A, Laricchia-Robbio L, Gianfaldoni D, Revoltella R, Del Bono G (2001) Comparison of a conventional immunoassay (ELISA) with a surface plasmon resonance-based biosensor for IGF-1 detection in cows' milk. Biosens Bioelectron 16(9–12): 971–977. 11679277 10.1016/s0956-5663(01)00245-7

[4] Gobi KV, Matsumoto K, Toko K, Ikezaki H, Miura N (2007) Enhanced sensitivity of self-assembled-monolayer-based SPR immunosensor for detection of benzaldehyde using a single-step multi-sandwich immunoassay. Anal Bioanal Chem 387(8): 2727–2735. 17318518 10.1007/s00216-007-1159-5

[5] Heutmekers TH, Bremer MG, Haasnoot W, Nielen MW (2007) A rapid surface plasmon resonance (SPR) biosensor immunoassay for screening of somatotropins in injection preparations. Anal Chim Acta 586(1–2): 239–245. 17386718 10.1016/j.aca.2006.11.047

[6] Hyuga M, Kawasaki N, Hyuga S, Ohta M, Itoh S, et al (2001) Rapid quantitation of follistatin by surface plasmon resonance (SPR) immunoassay. Kokuritsu Iyakuhin Shokuhin Eisei Kenkyusho Hokoku 119: 57–60. 11915286

[7] Indyk HE, Filonzi EL (2003) Determination of immunoglobulin G in bovine colostrum and milk by direct biosensor SPR-immunoassay. J AOAC Int 86(2): 386–393. 12723922

[8] Mytych DT, La S, Barger T, Ferbas J, Swanson SJ (2009) The development and validation of a sensitive, dual-flow cell, SPR-based biosensor immunoassay for the detection, semi-quantitation, and characterization of antibodies to darbepoetin alfa and epoetin alfa in human serum. J Pharm Biomed Anal 49(2): 415–426. 19135328 10.1016/j.jpba.2008.11.028

[9] Sun Y, Bai Y, Song D, Li X, Wang L, Zhang H (2007) Design and performances of immunoassay based on SPR biosensor with magnetic microbeads. Biosens Bioelectron 23(4): 473–478. 17764924 10.1016/j.bios.2007.06.016

[10] Wang J, Munir A, Li Z, Zhou HS (2009) Aptamer-Au NPs conjugates-enhanced SPR sensing for the ultrasensitive sandwich immunoassay. Biosens Bioelectron 25(1): 124–129. 19592231 10.1016/j.bios.2009.06.016

[11] Shriver-Lake LC, Donner B, Edelstein R, Breslin K, Bhatia SK, et al (1997) Antibody immobilization using heterobifunctional crosslinkers. Biosens Bioelectron 12(11): 1101–1106. 9451798 10.1016/s0956-5663(97)00070-5

[12] Schmid EL, Keller TA, Dienes Z, Vogel H (1997) Reversible oriented surface immobilization of functional proteins on oxide surfaces. Anal Chem 69(11): 1979–1985. 9183172 10.1021/ac9700033

[13] Anderson GP, Jacoby MA, Ligler FS, King KD (1997) Effectiveness of protein A for antibody immobilization for a fiber optic biosensor. Biosens Bioelectron 12(4): 329–336. 9178518 10.1016/s0956-5663(96)00074-7

[14] Lu B, Smyth MR, O'Kennedy R (1996) Oriented immobilization of antibodies and its applications in immunoassays and immunosensors. Analyst 121(3): 29R–32R. 10.1039/an996210029r 8729652

[15] Danczyk R, Krieder B, North A, Webster T, HogenEsch H, et al (2003) Comparison of antibody functionality using different immobilization methods. Biotechnol Bioeng 84(2): 215–223. 12966578 10.1002/bit.10760

[16] Vashist SK, Dixit CK, MacCraith BD, O'Kennedy R (2011) Effect of antibody immobilization strategies on the analytical performance of a surface plasmon resonance-based immunoassay. Analyst 136(21): 4431–4436. 21904732 10.1039/c1an15325k

[17] Rusmini F, Zhong Z, Feijen J (2007) Protein immobilization strategies for protein biochips. Biomacromolecules. Biomacromolecules 8(6): 1775–1789. 17444679 10.1021/bm061197b

[18] Ahluwalia A, De Rossi D, Ristori C, Schirone A, Serra G (1992) A comparative study of protein immobilization techniques for optical immunosensors. Biosens Bioelectron 7(3): 207–214. 1586474 10.1016/0956-5663(92)87017-j

[19] Steward MW, Lew AM (1985) The importance of antibody affinity in the performance of immunoassays for antibody. J Immunol Methods 78(2): 173–190. 2580911 10.1016/0022-1759(85)90074-2

[20] Nimmo GR, Lew AM, Stanley CM, Steward MW (1984) Influence of antibody affinity on the performance of different antibody assays. J Immunol Methods 72(1): 177–187. 6747301 10.1016/0022-1759(84)90446-0

[21] Liang M, Klakamp SL, Funelas C, Lu H, Lam B, et al (2007) Detection of high- and low-affinity antibodies against a human monoclonal antibody using various technology platforms. Assay Drug Dev Technol 5(5): 655–662. 17939757 10.1089/adt.2007.089

[22] Copley CG, Law B, Jenner WN (2002) Immunology and the production of reagent antibodies. In: Immunoassay a practical guide; Taylor & Francis.

[23] Kobayashi N, Oyama H, Kato Y, Goto J, Söderlind E, et al (2010) Two-step in vitro antibody affinity maturation enables estradiol-17beta assays with more than 10-fold higher sensitivity. Anal Chem 82(3): 1027–1038. 20047279 10.1021/ac902283n

[24] Muller BH, Savatier A, L'Hostis G, Costa N, Bossus M, et al (2011) In vitro affinity maturation of an anti-PSA antibody for prostate cancer diagnostic assay. Mol Biol 414(4): 545–562. 10.1016/j.jmb.2011.10.008 22019475

[25] Siegel RW, Baugher W, Rahn T, Drengler S, Tyner J (2008) Affinity maturation of tacrolimus antibody for improved immunoassay performance. Clin Chem 54(6): 1008–1017. 18403566 10.1373/clinchem.2007.097352

[26] Razai A, Garcia-Rodriguez C, Lou J, Geren IN, Forsyth CM, et al (2005) Molecular evolution of antibody affinity for sensitive detection of botulinum neurotoxin type A. J Mol Biol. 351(1): 158–169. 10.1016/j.jmb.2005.06.003 16002090

[27] Ehrlich PH, Moyle WR, Moustafa ZA, Canfield RE (1982) Mixing two monoclonal antibodies yields enhanced affinity for antigen. J Immunol 128(6): 2709–27013. 7077082

[28] Klonisch T, Panayotou G, Edwards P, Jackson AM, Berger P, et al (1996) Enhancement in antigen binding by a combination of synergy and antibody capture. Immunology 89(2): 165–171. 8943709 10.1046/j.1365-2567.1996.d01-722.x PMC1456500

[29] Ehrlich PH, Moyle WR, Moustafa ZA (1983) Further characterization of cooperative interactions of monoclonal antibodies. J Immunol 131(4): 1906–1912. 6619545

[30] Ehrlich PH, Moyle WR (1983) Cooperative immunoassays: ultrasensitive assays with mixed monoclonal antibodies. Science 221(4607): 279–281. 6857284 10.1126/science.6857284

[31] Bravman T, Bronner V, Lavie K, Notcovich A, Papalia GA, et al (2006) Exploring “one-shot” kinetics and small molecule analysis using the ProteOn XPR36 array biosensor. Anal Biochem 358(2): 281–288. 16962556 10.1016/j.ab.2006.08.005

[32] Surawska H, Ma PC, Salgia R (2004) The role of ephrins and Eph receptors in cancer. Cytokine Growth Factor Rev 15(6): 419–433. 15561600 10.1016/j.cytogfr.2004.09.002

[33] Meade-Tollin L, Martinez JD (2007) Loss of p53 and overexpression of EphA2 predict poor prognosis for ovarian cancer patients. Cancer Biol Ther 6(2): 288–289. 17426437 10.4161/cbt.6.2.4024

[34] Miyazaki T, Kato H, Fukuchi M, Nakajima M, Kuwano H (2003) EphA2 overexpression correlates with poor prognosis in esophageal squamous cell carcinoma. Int J Cancer 103(5): 657–663. 12494475 10.1002/ijc.10860

[35] Bradley SP, Hamer PJ, Carney WP (2010) Expression levels of circulating soluble-EphA2 receptor in cancer patients. Cancer Research 70(8), AACR 101st Annual Meeting. Abstract 826.

[36] Saphire EO, Stanfield RL, Crispin MD, Parren PW, Rudd PM, et al (2002) Contrasting IgG structures reveal extreme asymmetry and flexibility. J Mol Biol 319(1): 9–18. 12051932 10.1016/S0022-2836(02)00244-9

[37] Sandin S, Ofverstedt LG, Wikström AC, Wrange O, Skoglund U (2004) Structure and flexibility of individual immunoglobulin G molecules in solution. Structure 12(3): 409–415. 15016357 10.1016/j.str.2004.02.011

[38] Zeng C, Huang X, Xu J, Li G, Ma J, et al (2013) Rapid and sensitive detection of maize chlorotic mottle virus using surface plasmon resonance-based biosensor. Anal Biochem 440(1): 18–22. 23660014 10.1016/j.ab.2013.04.026

[39] Stenberg E, Persson B, Roos H, Urbaniczky C (1991) Quantitative determination of surface concentration of protein with surface plasmon resonance using radiolabeled proteins. Journal of Colloid and Interface Science 143(2): 513–526.

